# IS*26* Veers Genomic Plasticity and Genetic Rearrangement toward Carbapenem Hyperresistance under Sublethal Antibiotics

**DOI:** 10.1128/mbio.03340-21

**Published:** 2022-02-08

**Authors:** Da-Wei Wei, Nai-Kei Wong, Yuqin Song, Gang Zhang, Chao Wang, Juan Li, Jie Feng

**Affiliations:** a State Key Laboratory of Microbial Resources, Institute of Microbiology, Chinese Academy of Sciences, Beijing, China; b College of Life Science, University of Chinese Academy of Sciences, Beijing, China; c Clinical Pharmacology Section, Department of Pharmacology, Shantou University Medical College, Shantou, China; d State Key Laboratory of Infectious Disease Prevention and Control, National Institute for Communicable Disease Control and Prevention, Chinese Centers for Disease Control and Prevention, Changping, Beijing, China; Louis Stokes Veterans Affairs Medical Center

**Keywords:** antimicrobial resistance, *Klebsiella pneumoniae* carbapenemase (KPC), insertion sequence IS*26*, gene amplification, genomic plasticity, genetic rearrangement

## Abstract

Multidrug-resistant Gram-negative carriers of Klebsiella pneumoniae carbapenemases (KPCs) often subvert antibiotic therapy due to inadequate sensitivity in laboratory detection. Although unstable gene amplification has been recognized to crucially contribute to underestimation or misestimation of antimicrobial resistance in clinical isolates, the precise mechanisms underlying carbapenem resistance driven by amplification of *bla*_KPC-2_ remain obscure. Here, we reported that IS*26*-mediated amplification of *bla*_KPC-2_ rapidly and robustly gave rise to carbapenem hyperresistant phenotypes in an Escherichia coli clinical strain following sublethal meropenem or tobramycin preexposure. Intriguingly, IS*26* also underpinned amplification of a 47 kb multiple drug resistance (MDR) region encompassing nine antibiotic resistance genes and six IS*26* insertion sequences. Tandem-repeat analysis and experimental validation demonstrated that *bla*_KPC-2_ amplification was indeed mediated by IS*26*, which was further experimentally shown to involve intricate genetic rearrangement. Such gene amplification arose dynamically under antibiotic stress and subsided upon antibiotic withdrawal. Instead of reducing the amplification of the IS*26*-flanked MDR region, drug combinations *in vitro* exacerbated it. Our study, thus, provides valuable insights into how dynamic gene amplification processes can precipitously transform resistance status and complicate diagnosis.

## INTRODUCTION

The global spread of carbapenem-resistant Enterobacteriaceae poses a relentless menace to public health systems, necessitating urgent concerted actions to innovate strategies for antimicrobial control and treatment (1). Members of Enterobacteriaceae develop carbapenem resistance primarily through the acquisition of carbapenemases with Klebsiella pneumoniae carbapenemases (KPCs) being the most prevalent (2). Typically, multidrug-resistant and recalcitrant to treatment Enterobacteriaceae KPC-producers have disseminated worldwide and become a major cause of increased morbidities and mortalities in healthcare-associated infections (HAIs) in hospital niches (3). Additionally, KPC-producing opportunistic pathogens have gained notoriety for subverting antibiotic therapy (4, 5) because they often exhibit deceptively low-level resistance to carbapenems in routine laboratory screens, leading to misdiagnosis or underdiagnosis (3, 6). For example, automatic detection systems reportedly identified up to 87% of KPC-producing K. pneumoniae (KPC-KP) as being susceptible to imipenem or meropenem (2). Intrinsically, the difficulty of reliably detecting antimicrobial susceptibility of KPC-KP isolates to carbapenems is due in part to heteroresistance, wherein a subpopulation with greater resistance to antibiotic stress than a dominant population is present, while the proportion of the former gradually rises in the presence of antibiotics (7). Gene amplification has been proposed as one of the drivers of heteroresistance. Notably, amplified *bla*_KPC_ genes have been detected in K. pneumoniae isolates with profound implications for the reduction of pathogens’ susceptibility to carbapenems (8, 9). To this date, however, the precise mechanisms underlying carbapenem resistance mediated by *bla*_KPC-2_ amplification, particularly from the perspective of carbapenem heteroresistance contributed by unstably amplified *bla*_KPC_, remain elusive.

Conventionally, clinical use of antibiotics is guided by both empirical experience and laboratory findings whose rationale is to attain the best cure rates with the highest possible nontoxic drug concentrations without provoking the development of *de novo* resistance within patients (10). In practice, due to a constant gap in diagnostic precision, many human and animal treatment regimens have proved insufficient for achieving such goals because pharmacologically effective antibiotic concentrations often fall below laboratory-determined MICs in body compartments and tissues *in vivo*, resulting in suboptimal inhibition of target bacteria. Indeed, growing evidence demonstrates that sublethal concentrations of antibiotics are a significant driver for the development of antibiotic resistance (11, 12). Sublethal, subinhibitory concentrations of antibiotics refer to those below MICs. Recent studies have shown that low-level antibiotic concentrations exert their effects basically on three different levels as selectors of resistance, generators of genetic and phenotypic variability, and signaling molecules (10). However, the molecular underpinnings of how sublethal antibiotics drive the processes of heteroresistance formation remain to be elucidated.

Here, our investigation focused on an E. coli clinical strain carrying the *bla*_KPC-2_ gene, which was isolated from a fatal case of a patient who failed to respond to antibiotic treatment. After exposure to a sublethal dose of carbapenem (2 μg/mL) for 24 h, the strain rapidly and robustly adapted to a range of high-level carbapenem doses during regrowth, accompanied by amplification of *bla*_KPC-2_. This amplification was positively associated with an extraordinary increase in antimicrobial resistance (AMR) as exemplified by amplified resistance determinants, including a 47 kb multiple drug resistance (MDR) region flanked by IS*26*. We monitored the dynamics of genomic plasticity in terms of copy number of amplified gene elements as well as any rearrangement of the MDR region. Tandem-repeat analysis and experimental validation demonstrated that *bla*_KPC-2_ amplification was mediated by IS*26.* Remarkably, combination therapy of meropenem (a carbapenem) and tobramycin (an aminoglycoside) failed to reduce amplification of the IS*26*-flanked MDR region harboring resistance genes but instead aggravated its progression. Our present work thus fills a critical gap in our understanding of IS*26*-driven amplification of *bla*_KPC-2_ as a driving force of AMR through which Enterobacteriaceae bacteria manage to evade carbapenem assaults, and thus compromise the efficacy of antibiotics in clinical use.

## RESULTS

### Preexposure to sublethal antibiotics rapidly unfetters carbapenem hyperresistance.

In our previous study, three outbreak-associated strains of E. coli (sequence type ST131) were separately isolated from sputum, urine, and blood samples collected within 1 month from a patient (13). These strains exhibited low-level carbapenem resistance (MIC for meropenem: 4 μg/mL; and MIC for biapenem: 4 μg/mL) and shared nearly identical genomic sequences, indicating that they likely arose from a single parent clone. Subsequent whole-genome analysis showed that a plasmid (pE0171; GenBank accession no. MK370988) carrying *bla*_KPC-2_, which conferred carbapenem resistance, was present in the isolates. Notably, *bla*_KPC-2_ was in an IS*26*-based composite transposon (Unit 1), whereas two copies of Unit 1 were found in the MDR region (Fig. S1). During antiinfective management, the patient had been treated with biapenem at 0.6 g per day for 15 days. Nonetheless, biapenem therapy proved ineffective and the patient later died of complications from E. coli infection despite a reported peak plasma concentration of biapenem (9.49 to 25.81 μg/mL) greater than 4 μg/mL (14). Based on observations of this clinical course of the disease, we hypothesized that a sustained low concentration of carbapenem *in vivo* could lead to detrimental development of resistance.

10.1128/mbio.03340-21.1 FIG S1 Diagram on the structure of MDR region in the plasmid. Download FIG S1, EPS file, 0.1 MB. Copyright © 2022 Wei et al. 2022 Wei et al. https://creativecommons.org/licenses/by/4.0/ This content is distributed under the terms of the Creative Commons Attribution 4.0 International license.

**FIG 1:**
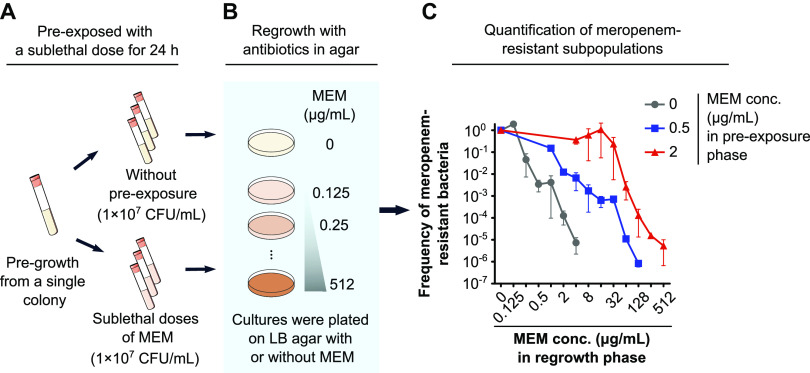
Exposure to sublethal antibiotics rapidly unleashed carbapenem hyperresistance. (A) Preexposure with or without a sublethal dose of meropenem (0.5 and 2 μg/mL) in broth for 24 h from a single colony of E. coli E01-7-1. (B) Cultures preexposure with or without sublethal dose meropenem were reexposed on LB agar with a gradient of meropenem concentrations (0 to 512 μg/mL). (C) Quantification of the meropenem-resistant subpopulation. These experiments were independently repeated 3 times and the error bars represent the standard error of the mean (*n *= 3).

For experimental proof of concept, one of the above-mentioned E. coli clinical isolates, E01-7-1, was selected for further experimentation. Broth containing 0, 0.5, or 2 μg/mL of meropenem was inoculated with the strain followed by culture for 24 h (Fig. 1A). The inoculums were then taken and subcultured individually on agar plates containing a gradient of meropenem concentrations (0 to 512 μg/mL) for regrowth (Fig. 1B). The percentage of bacteria exhibiting meropenem (MEM) resistance was calculated as the number of colonies grown on LB agar plates containing MEM divided by the number of bacterial colonies grown on plain LB agar plates. Our results showed that the highest concentration of meropenem permissive to Fithe growth of the control bacteria (i.e., without preexposure to sublethal antibiotics) was 4 μg/mL, while the frequency of meropenem (MEM)-resistant bacteria was about 7.35 × 10^−6^ (Fig. 1C). In comparison, the highest growth-permissive meropenem concentration was found to be 128 μg/mL and 512 μg/mL for groups preexposed to sublethal meropenem at 0.5 and 2 μg/mL, respectively, and the corresponding frequencies of meropenem-resistant subpopulations on plates were determined to be 8.45 × 10^−7^ and 4.63 × 10^−6^, respectively. The frequency of resistant bacteria declined evidently with increasing antibiotic concentration. Strikingly, however, the frequency of resistant bacteria in groups preexposed to 2 μg/mL meropenem and then regrown on meropenem containing agar plates (8, 16, and 32 μg/mL) approached 1. This suggested that the presence of sublethal meropenem concentration (2 μg/mL) was sufficient and potent to cause the emergence of meropenem-resistant subpopulations with exceedingly high-level resistance within a relatively short time (e.g., red line in Fig. 1C).

When bacterial samples preexposed to sublethal meropenem (2 μg/mL, 24 h) were added as an inoculum to broth medium containing meropenem (4 to 512 μg/mL) for regrowth, results similar to those of regrowth in agar plates were obtained (Table S1). Broths inoculated with sublethal antibiotic preexposed bacteria were observed to allow growth in the presence of 512 μg/mL meropenem. In contrast, broths inoculated with unexposed bacteria showed bacterial regrowth in a medium containing hardly more than 4 μg/mL meropenem during regrowth. Interestingly, the efflux pump inhibitor phenylalanine-arginine-*β*-naphthylamide dihydrochloride (PA*β*N; 50 μM), had no effects on meropenem MIC (Table S1), suggesting that efflux pumps had little or no contribution to phenotypic outcomes in MICs. These results indicated that sublethal antibiotic concentrations favored the enrichment of bacterial subpopulations that were selectively at an advantage in acquiring resistance.

10.1128/mbio.03340-21.4 TABLE S1 Minimum inhibitory concentrations (MIC) of E01-7-1 for meropenem. Download Table S1, XLSX file, 0.01 MB. Copyright © 2022 Wei et al. 2022 Wei et al. https://creativecommons.org/licenses/by/4.0/ This content is distributed under the terms of the Creative Commons Attribution 4.0 International license.

### Sublethal meropenem preexposure elicits hyperresistance via dynamic processes of gene amplification.

**FIG 2:**
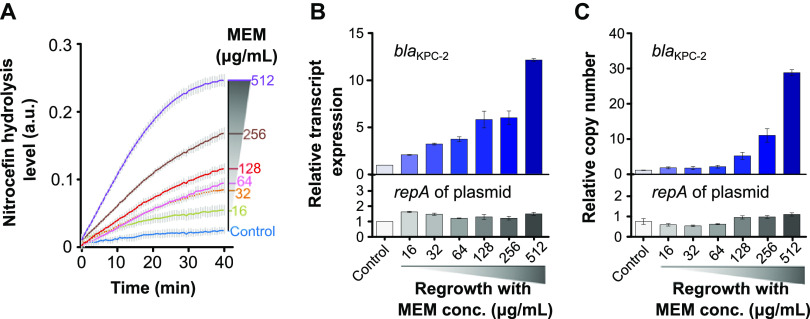
An increase in copy number of the *bla*_KPC-2_ gene under carbapenem stress underpinned hyperproduction of KPC. (A) Nitrocefin hydrolysis activity in strains reexposed to various concentrations of meropenem. (B) Expression of *bla*_KPC-2_ and *repA* (encoding replication protein of the plasmid) relative to the housekeeping gene *purA* in strains exposed to various concentrations of meropenem. (C) Copy numbers of *bla*_KPC-2_ and *repA* relative to *purA* in strains exposed to various concentrations of meropenem. These experiments were independently repeated 3 times and the error bars represent the standard error of the mean (*n *= 3).

To scrutinize this phenomenon, we analyzed the sequences of carbapenem resistance genes (*bla*_KPC-2_, *ompC*, and *ompF*) and their promoter regions in strains grown with or without stress from sublethal meropenem and in strains regrown with high-dose meropenem. No mutations were detected among the strains. However, an increase of 1.6-fold in copy number of *bla*_KPC-2_ under sublethal meropenem (2 μg/mL) relative to untreated conditions was observed. To better understand the mechanism permitting regrowth in high-dose meropenem, we determined the production of *β*-lactamases, expression of *bla*_KPC-2,_ and copy number of *bla*_KPC-2_ in bacterial cells exposed to high concentrations of meropenem (ranging from 16 to 512 μg/mL) following sublethal meropenem preexposure (2 μg/mL). Remarkably, *β*-lactamase levels rose with increasing concentrations of antibiotics (Fig. 2A). Similarly, the qRT-PCR results showed statistically significant increases in expression of the *bla*_KPC-2_ gene, although without an increase in *repA* of plasmid pE0171, which was a carrier of *bla*_KPC-2_ (Fig. 2B; original data in Table S2). Finally, we confirmed that the copy number of *bla*_KPC-2_ was positively correlated with antibiotic concentration (ranging from 16 to 512 μg/mL) with respect to the normalized levels of the housekeeping gene *purA* encoding adenylosuccinate synthase (Fig. 2C). The relative copy number of *bla*_KPC-2_ was 1.2 in the absence of meropenem stress, which dramatically rose to 28.9 at 512 μg/mL meropenem (Fig. 2C; data in Table S2). These observations strongly suggested that an augmentation in *bla*_KPC-2_ copy number could account for the rapid increase in resistance to meropenem. Predictably, the copy number of *repA* was unaltered, implying that amplification of *bla*_KPC-2_ was not tied to an increase in the copy number of the plasmid carrying *bla*_KPC-2_. This novel finding lends support to the notion that rapid emergence of resistance could sequentially arise from preexposure to a single sublethal-dose antibiotic (such as meropenem) and subsequent reexposure to antibiotics at higher concentrations.

10.1128/mbio.03340-21.5 TABLE S2 Mean and standard error of the mean of qPCR data presented in this study. Download Table S2, XLSX file, 0.02 MB. Copyright © 2022 Wei et al. 2022 Wei et al. https://creativecommons.org/licenses/by/4.0/ This content is distributed under the terms of the Creative Commons Attribution 4.0 International license.

### Genomic plasticity through amplification of an IS*26*-flanked MDR region with *bla*_KPC-2_ and complex genomic arrangements.

**FIG 3:**
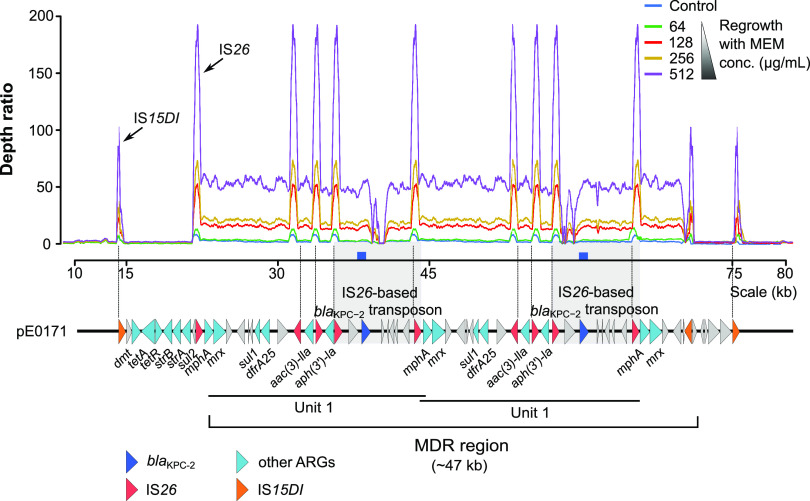
Read mapping depths of the MDR region relative to that of housekeeping *purA* in strains reexposure to meropenem. Different colors represent antibiotic concentrations.

To dissect the mechanisms underlying amplification of *bla*_KPC-2_ genes, we applied Illumina sequencing to the analysis of DNA extracted from strains subjected to various levels of meropenem stress at the end of the logarithmic phase during regrowth (clean data size and mean coverage as shown in Table S3). We then mapped short reads onto the complete genome of the strain E01-7-1. Analysis on read-mapping depths relative to the housekeeping gene *purA* revealed a 47 kb MDR region containing *bla*_KPC-2_ with significantly higher coverage of reads in strains exposed to meropenem relative to untreated control strains (Fig. 3 and Table S3). The read-mapping depth ratio of this region was positively associated with antibiotic concentration with changes in *bla*_KPC-2_-mapping depth being 1.63-fold higher in the meropenem preexposed (2 μg/mL) strains and 1.96 to 29.94-fold higher in the meropenem regrowth (64 to 512 μg/mL) stains than in the case where bacteria were unexposed. The aforementioned 47 kb region turned out to be flanked by IS*26* and IS*15DI*, which share 99% nucleotide identity and 100% coverage with IS*26*. Changes in IS*26*-mapping depths were found to increase correspondingly by 1.28 to 21.12-fold. Furthermore, the amplified region contained 16 antibiotic resistance genes (ARGs) underpinning resistance to macrolides, aminoglycosides, carbapenems, sulfonamides, and diaminopyrimidines. Considering the existence of two Unit 1 sequences, 7 types of ARGs were identified. The results implicate IS*26* as a prominent mediator of amplification of *bla*_KPC-2_ genes during the rapid emergence of hyperresistance following sublethal antibiotic challenges. Furthermore, unexpected amplification of the MDR region under single-drug stress underscored the complexity of such gene amplification.

10.1128/mbio.03340-21.6 TABLE S3 Clean data size, mean coverage, average of mapping depth, and depth ratio relative to *purA* and *repA* from the Illumina short reads in this study. Download Table S3, XLSX file, 0.02 MB. Copyright © 2022 Wei et al. 2022 Wei et al. https://creativecommons.org/licenses/by/4.0/ This content is distributed under the terms of the Creative Commons Attribution 4.0 International license.

**FIG 4:**
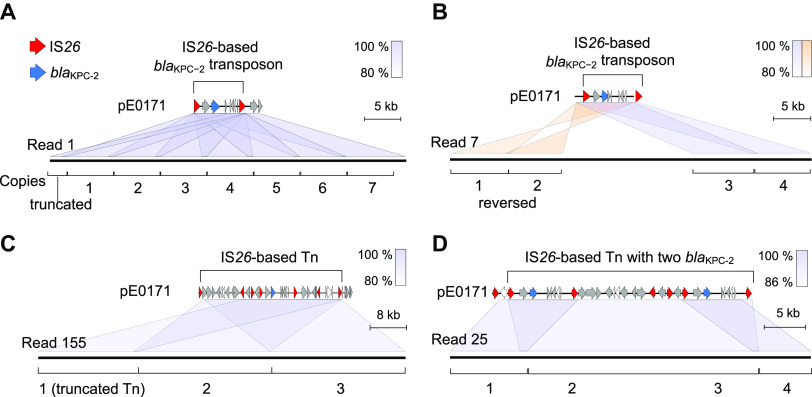
Gene organization of long reads containing multiple copies of *bla*_KPC-2,_ as revealed by Nanopore sequencing. (A) Seven identical *bla*_KPC-2_ transposons in a tandem array in read 1. (B) Two direct and two inverted *bla*_KPC-2_ transposons linked together in read 7. (C) Three tandem units, each harboring nine ARGs and six IS*26*s, bordered by two IS*26s* in read 155. (D) Amplification of a composite transposon with *bla*_KPC-2_ flanked by IS*26*. Red triangles denote IS*26*, blue triangles denote *bla*_KPC-2_, and gray triangles denote other genes.

To advance a more detailed mechanistic picture of the *bla*_KPC-2_ amplification involved, we applied Oxford Nanopore Technologies (ONT) sequencing to the acquisition of long reads of the E. coli strain exposed to 512 μg/mL meropenem. In total, 5,295 reads containing *bla*_KPC-2_ were obtained, among which 1,287 reads indicated carriage of more than two copies of *bla*_KPC-2_ (Table 1). An unexpected level of diversity in *bla*_KPC-2_ copy numbers was observed among the reads, which underlined the complexity of the gene amplification machinery. To illustrate, the amplification of *bla*_KPC-2_ possibly occurred through two processes (Table 1) with the first being tandem IS*26*-based *bla*_KPC-2_ transposon amplification. For example, seven identical *bla*_KPC-2_ transposons in a tandem array were found within a 59 kb read (Fig. 4A). In addition to direct repeats in tandem, two inverted *bla*_KPC-2_ transposons linked together were present in read 7 (Fig. 4B). The second mechanism presumably occurred via amplification of a composite transposon with *bla*_KPC-2_ flanked by IS*26.* For instance, read 155 was composed of three 31 kb tandem units bordered by two IS*26*s with nine ARGs and six IS*26*s in each unit (Fig. 4C). Read 25 likely included tandem units containing two *bla*_KPC-2_ copies (Fig. 4D). These read data further confirmed that amplification of *bla*_KPC-2_ was mediated by IS*26*. Accordingly, we analyzed the copy number of IS*26* transposase by qPCR. In searching for a possible correlation between copy number and transcription level of *bla*_KPC-2_ and IS*26* genes, it was found that the copy number of IS*26* and *bla*_KPC-2_ (*r *= 0.92) and the transcription level of IS*26* and *bla*_KPC-2_ (*r *= 0.97) were strongly correlated (Fig. S2; original data in Table S2). Of note, these results showed a trend similar to that of *bla*_KPC-2_ under antibiotic stress but with higher copy numbers of IS*26* transposase, which is consistent with the E. coli genome possessing more basal copies of the genes (12 copies).

**TABLE 1:**
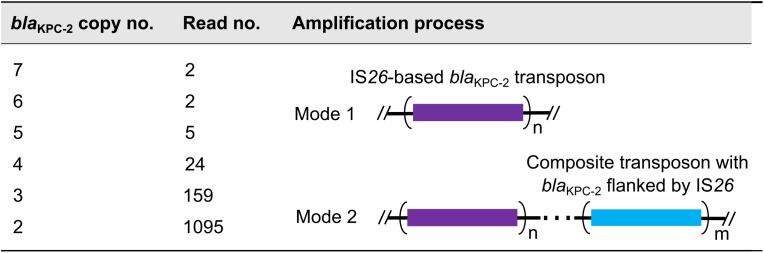
Analysis on blaKPC-2 copy number in reads^*a*^

a Amplification of *bla*_KPC-2_ occurs in two ways: tandem amplification of IS*26*-based *bla*_KPC-2_ transposon (Mode 1), and amplification of a composite transposon with *bla*_KPC-2_ flanked by IS*26* (Mode 2), as detailed in Fig. 4.

10.1128/mbio.03340-21.2 FIG S2 Analysis on the correlation between copy number and transcript level of blaKPC-2 and IS*26*. For instance, a Pearson's correlation coefficient of r = 0.92 indicates that the correlation between the relative copy numbers of blaKPC-2 and IS*26* is relatively strong. Download FIG S2, TIF file, 0.3 MB. Copyright © 2022 Wei et al. 2022 Wei et al. https://creativecommons.org/licenses/by/4.0/ This content is distributed under the terms of the Creative Commons Attribution 4.0 International license.

By leveraging a hybrid assembly of ONT long-sequencing reads and Illumina short-sequencing reads, we obtained a complete genome of strain E01-7-1 cultured in the presence of 512 μg/mL meropenem. The assembled genome was 5.1 Gb in length with a GC content of 50.89% and contained a 137 kb plasmid. In contrast, there was again no *bla*_KPC-2_ amplification mediated by IS*26* found in the MDR region of the plasmid. This intriguing finding suggested that strain E01-7-1 could indeed generate subpopulations with multiple copies of *bla*_KPC-2_ upon carbapenem, but these subpopulations may carry diverse structural variants of the MDR region or may not be dominant in the population. Phenotypically, strains with such properties were deemed to exhibit heteroresistance. Furthermore, the circular genomic DNA molecule did not appear to contain an IS*26*-mediated translocatable unit (TU).

### Experimental validation of *bla*_KPC-2_ amplification mediated by IS*26*.

**FIG 5:**
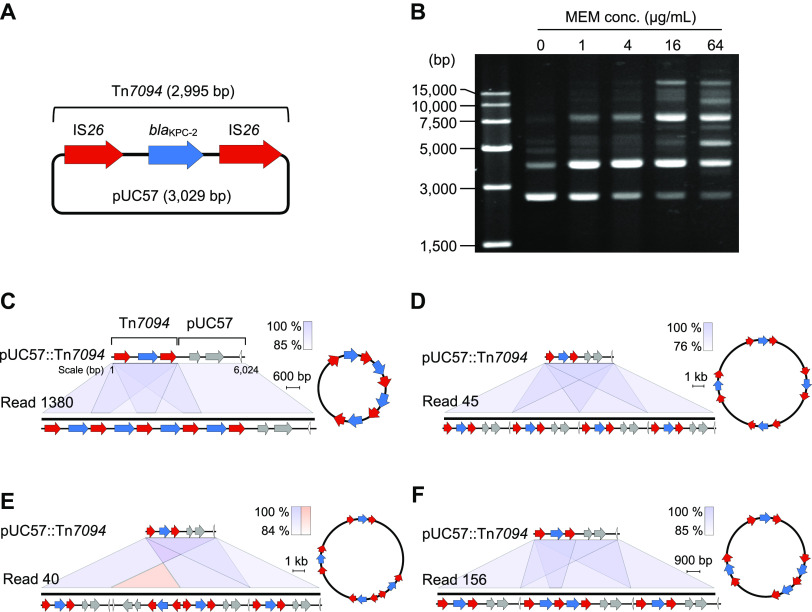
Analysis on dynamics of Tn*7094* evolution in E. coli strains preexposed and reexposed to various concentrations of meropenem. (A) Structural design of plasmid pUC57:Tn*7094*. (B) Plasmid profiles analysis on the dynamics of Tn*7094* evolution in strains exposed to 0 μg/mL, 1 μg/mL, 16 μg/mL, or 64 μg/mL meropenem, respectively. (C) Gene organization of long reads containing four tandem arrays of Tn*7094* on the plasmid as revealed by Nanopore sequencing. (D) Tandem array of the plasmid pUC57:Tn*7094* in a direct-repeat configuration. (E) Tandem array of the plasmid pUC57:Tn*7094* in direct and inverted repeat configurations. (F) Composite structure from combined amplification of Tn*7094* and the plasmid.

To experimentally interrogate the roles of IS*26* in amplification of *bla*_KPC-2_, we constructed a plasmid consisting of the vector pUC57 and IS*26*-*bla*_KPC-2_-IS*26* (Fig. 5A). The IS*26*-*bla*_KPC-2_-IS*26* sequence was designated Tn*7094* (∼2.9 kb) (LSTM, https://transposon.lstmed.ac.uk/tn-registry). Next, E. coli Top10 (*recA* deficiency) was transformed with the pUC57:Tn*7094* plasmid by using kanamycin and meropenem as selective markers. In observation, the recombinant plasmid intensified the resistance of its E. coli host against meropenem, from < 0.25 to 2 μg/mL. Subsequently, an inoculum (1 × 10^7^ colony forming unit [CFU]) of E. coli with the recombinant plasmid was incubated in a liquid medium containing 1 μg/mL meropenem (or 1/2 MIC) for 24 h. These sublethal antibiotic preexposed bacterial cells were then challenged with various concentrations of meropenem (ranging from 0 to 64 μg/mL) during the regrowth phase. To monitor the dynamics of Tn*7094* under various levels of meropenem stress, plasmids were extracted from culture. Analysis on plasmid patterns corroborated that more bands of higher molecular weights were detectable in extracts from bacterial cells incubated at higher levels of meropenem compared with that at 0 μg/mL, which could be a direct consequence of gene amplification mediated by IS*26* (Fig. 5B).

As a next step, we conducted Nanopore sequencing to characterize the amplification of Tn*7094*. Plasmids from strains grown at 64 μg/mL meropenem were extracted and subjected to Nanopore sequencing analysis. The numbers of reads containing pUC57:Tn*7094* and pUC57:IS*26* were 121,166 and 113,722, respectively. Amplification of Tn*7094* was found in 3,820 reads, which corresponded to the carriage of at least two *bla*_KPC-2_ sequences (Table 2). We reasoned that such amplification may take place via three mechanisms, namely, tandem Tn*7094* amplification (Fig. 5C and Table 2), plasmid amplification, in which the plasmid pUC57:Tn*7094* was aligned in a direct or inverted repeat configuration (Fig. 5D and E), and amplification of Tn*7094* and a plasmid of the composite structure (Fig. 5F). Overall, in addition to Tn*7094* amplification being mediated by IS*26*, we also observed intricate IS*26*-mediated multimerization of plasmids. Nanopore sequencing confirmed our results for plasmid analyses and provided detailed sequence information for elucidating the dynamics of IS*26*-mediated amplification (Table 2).

**TABLE 2:**
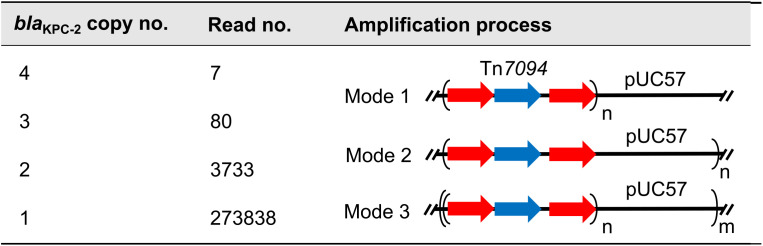
Analysis on blaKPC-2 amplification in pUC57: Tn7094^*a*^

a Amplification of *bla*_KPC-2_ occurs in three ways: amplification of tandem Tn*7094* (Mode 1), tandem amplification of the plasmid pUC57: Tn*7094* (Mode 2), and a composite structure that combines amplification of Tn*7094* and the plasmid (Mode 3). For further details, see Fig. 5.

### Amplification of IS*26*-flanked MDR region with *bla*_KPC-2_ is unstable.

**FIG 6:**
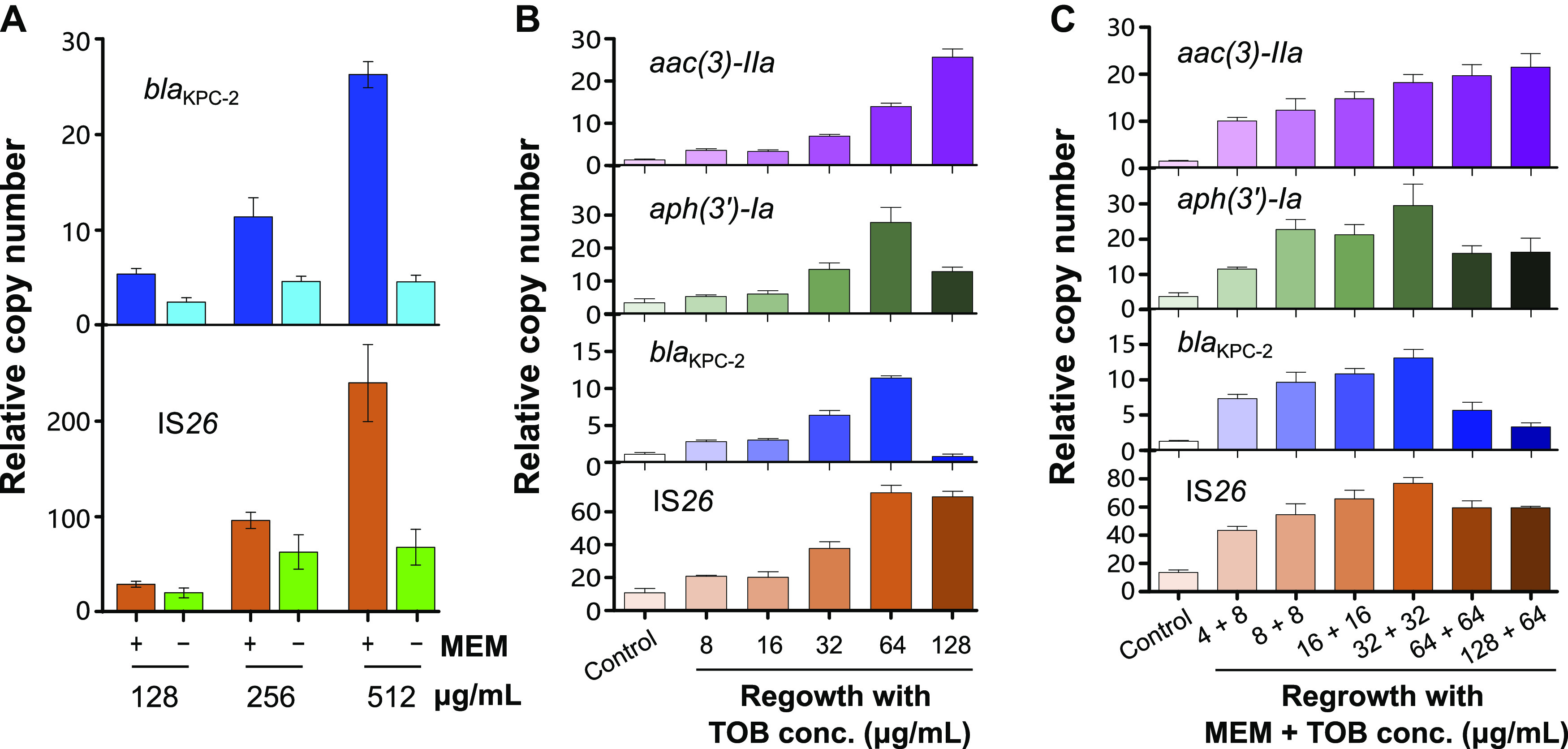
Copy number instability and the effect of preexposure and reexposure to other antibiotics on genes copy number. (A) Comparison of relative copy numbers of *bla*_KPC-2_ and IS*26* in strains during antibiotic exposure and subsequent withdrawal. “+” refers to a strain cultured with corresponding concentration (128 μg/mL, 256 μg/mL, and 512 μg/mL) of meropenem followed by antibiotic-free medium (“-”) for 24 h. (B) Relative copy numbers of *aac(3)-IIa*, *aph(3′)-Ia*, *bla*_KPC-2_, and IS*26* relative to that of *repA* in strains preexposed and reexposed to tobramycin. (C) Relative copy numbers of *aac(3)-IIa*, *aph(3′)-Ia*, *bla*_KPC-2_, and IS*26* relative to that of *repA* in strains preexposed and reexposed to tobramycin and meropenem. These experiments were independently repeated 3 times and the error bars represent the standard error of the mean (*n *= 3).

Because gene amplification typically results in protein hyperproduction, we intuitively conjectured that this process imposed a fitness cost, at least initially, on the bacterium in response to a forced increase in metabolic activities (15). Accordingly, we assessed the stability of amplified genes in the absence of any antibiotic stress. After transferring a strain E01-7-1 previously exposed to high concentrations of meropenem (128, 256, or 512 μg/mL) back to fresh medium without antibiotics and further culturing it for 24 h, the *bla*_KPC-2_ and IS*26* copy numbers fell sharply relative to that of *repA* (Fig. 6A; original data in Table S2). Specifically, upon antibiotic withdrawal, the copy numbers of *bla*_KPC-2_ decreased by 2.2, 2.5, or 5.8-fold in the cultural medium compared to groups exposed to 128, 256, or 512 μg/mL meropenem, respectively. Similarly, the copy numbers of IS*26* also dropped by 1.4, 1.6, or 3.5-fold, respectively. Further Illumina sequencing analysis demonstrated that the 47 kb MDR region, which was rapidly amplifiable during antibiotic treatment, declined in copy number after 24 h of growth in a medium free of meropenem (Fig. S3A and Table S3). Collectively, these results demonstrated that amplification of these genetic elements was unstable, resulting in regression to phenotypes of lower copy numbers in the absence of antibiotic stress. This agrees with the notion that bacteria tend to maximize their survival by responding to environmental cues and ingeniously switching their gene expression programs in a rapid, resource-limiting manner.

10.1128/mbio.03340-21.3 FIG S3 Read mapping depths of the MDR region. (a) Depth ratio relative to purA in strains during meropenem exposure and subsequent antibiotic withdrawal. (b) Depth ratio relative to repA in strains reexposed to various concentrations of tobramycin. (c) Depth ratio relative to repA in strains reexposed under the challenge of various concentrations of meropenem and tobramycin. Different colors represent antibiotic concentrations. Download FIG S3, TIF file, 0.4 MB. Copyright © 2022 Wei et al. 2022 Wei et al. https://creativecommons.org/licenses/by/4.0/ This content is distributed under the terms of the Creative Commons Attribution 4.0 International license.

### Amplification of the MDR region also occurred in response to tobramycin stress.

According to genome sequencing analysis, six types of ARGs along with *bla*_KPC-2_ were located within the MDR region. Two of these, *aac(3)-IIa* and *aph(3′)-Ia*, are known to be resistance determinants for aminoglycosides and were found to be flanked by IS*26*. A logical question that followed was whether *bla*_KPC-2_ amplification can occur under stress from preexposure to antibiotic classes other than *β*-lactams or carbapenems. To this end, we incubated the E. coli strain E01-7-1 in liquid medium containing 8 μg/mL tobramycin (or 1/2 MIC) for 24 h. We then inoculated the medium with antibiotic pretreated bacterial cells in the presence of various concentrations of tobramycin (from 16 to 128 μg/mL). Strikingly, the cells could grow well at 128 μg/mL tobramycin during regrowth. The copy numbers of *aac(3)-IIa*, *aph(3′)-Ia*, *bla*_KPC-2_, and IS*26* increased relative to that of *repA* in response to a gradient of antibiotic concentrations. However, the copy numbers of *aph(3′)-Ia* and *bla*_KPC-2_ decreased at 128 μg/mL tobramycin (Fig. 6B; original data in Table S2). These results suggested that unequal amplification of a unit flanked by IS*26* and another unit carrying *aph(3′)-Ia* most likely contributed to survival advantages against tobramycin stress. Illumina sequencing confirmed our results for the qPCR assays in which the read-mapping depths of the MDR region relative to that of *repA* increased with antibiotic concentration except for the *bla*_KPC-2_ transposon, which exhibited a marked decrease at 128 μg/mL tobramycin (Fig. S3B; original data in Table S3). Collectively, we furnished the first evidence that preexposure to tobramycin instead of meropenem was also sufficient to trigger amplification of the MDR region, which harbored multiple ARGs other than *bla*_KPC-2_.

### Drug combination *in vitro* fails to check amplification of MDR region with *bla*_KPC-2_.

Conventionally, combinations of multiple antibiotics have been used in clinical practice to slow the emergence of AMR with some success (16). Therefore, we asked whether a combination of antibiotics could help retard or halt the amplification of the MDR region. To this end, E. coli strain E01-7-1 was cultured in a liquid medium containing 8 μg/mL tobramycin and 2 μg/mL meropenem for 24 h. Subsequently, the pretreated cells were subcultured correspondingly in the presence of various antibiotic concentrations. The relative copy numbers of *aac(3)-IIa*, *aph(3′)-Ia*, *bla*_KPC-2_, and IS*26* were determined to be 18.22, 29.55, 13.08, and 76.76, respectively, at 32 μg/mL tobramycin and 32 μg/mL meropenem, which were higher than those for exposure to single drugs at the same concentrations (Fig. 6C; original data in Table S2). The copy numbers of *aph(3′)-Ia*, *bla*_KPC-2_, and IS*26* decreased at higher levels of antibiotic stress while that of *aac(3)-IIa* continued to rise. Evidence from Illumina sequencing confirmed our results for qPCR (Fig. S3C and Table S3). These observations indicated that combination therapy not only failed to retard amplification of resistance genes flanked by IS*26* but also escalated IS*26*-driven amplification to a greater extent than any of the monotherapy drugs tested during antibiotic preexposure.

## DISCUSSION

Sublethal or subinhibitory concentrations of antibiotics do commonly occur *in vivo* in clinical treatment contexts, which provided a basis for the evolution of adaptively resilient clones exhibiting heteroresistance (17). *bla*_KPC-2_ has been recognized as an alarming contributor to carbapenem resistance among Gram-negative pathogens worldwide (3, 18–20). Heteroresistant KPC-producers are highly challenging to detect in the laboratory due to their apparent yet deceptively low levels of carbapenem resistance (3, 5, 19). In this study, we provide the first detailed evidence for IS*26*-mediated amplification of *bla*_KPC-2_ as a driving force for the rapid emergence of carbapenem hyperresistance in E. coli, whose intensity is strongly coupled to antibiotic stress following sublethal dose preexposure. However, this amplification is unstable and transient, adding to the difficulty in clinical identification.

In terms of mechanisms, our results demonstrated that IS*26*-dependent amplification of *bla*_KPC-2_ rapidly drives the emergence of carbapenem hyperresistance following preexposure to sublethal antibiotics, implying that bacterial subpopulations with amplified *bla*_KPC-2_ must have been enriched or induced under a low or sublethal concentration of meropenem (or even other antibiotics) to effectively counter higher-level antimicrobial assaults. In addition, copy numbers of *bla*_KPC-2_ and the IS*26* transposon were linked to the strength of antibiotic stress. In the presence of antibiotic-induced selective pressure, resistant bacterial cells endowed with amplification capacities became progressively enriched, whereas, in the absence of antibiotics, susceptible bacterial cells with lower copy numbers acquired a growth advantage. This growth pattern is characteristic of heteroresistance (15). As sublethal antibiotic concentrations can naturally occur *in vivo* in some pharmacodynamic contexts and across tissues of different depths among patients and livestock (12), the phenomenon reported herein could partially account for the failure of antimicrobial intervention against KPC-producers which exhibit deceptively low-level resistance to carbapenems in routine laboratory screens.

It is noteworthy that in addition to *bla*_KPC-2,_ many other resistance genes have been found on compound transposons bounded by IS*26*, while MDR regions often include multiple IS*26* transposons containing several ARGs (21–23). In our study, we compellingly demonstrated efficient amplification of an MDR region containing 16 ARGs mediated by IS*26* in response to a single antibiotic or a combination of antibiotics. Previously, evidence has shown that combinatorial use of antibiotics can effectively inhibit amplification caused by a single antibiotic, supporting the wisdom of empirical practice (16). However, our present study has demonstrated situations wherein a drug combination *in vitro* not only failed to halt but exacerbated IS*26*-mediated amplification of the MDR region. Therefore, we reason that combination therapy may not be universally applicable to all cases of bacterial infections and that greater caution is needed for its use against HAIs caused by multidrug-resistant strains armed with multiple IS*26* transposons because insufficiently informed combination therapies may carry their risks of inadvertently complicating resistance development.

Through analysis of long reads, our study also shed new light on the intricate nature of *bla*_KPC-2_ amplification. Multiple IS*26* sequences are present in the MDR region of the clinical strain E01-7-1, and various amplified units flanked by IS*26* were recomposed from this diverse MDR region landscape during amplification. In E. coli carrying the constructed plasmid pUC57:Tn*7094* and amplification of Tn*7094*, the plasmid became cointegrated through homologous recombination, resulting in multiple copies. These data suggest a crucial role of IS*26* in mediating MDR region activity and plasmid evolution under selective pressure. The amplified units were frequently rearranged in a tandem array. Consistent with our observations, a recent study showed that tandem amplification of *bla*_TEM-1B_ is achieved through excision of a TU from an IS*26*-flanked transposon and reinsertion into an adjacent IS*26* (24). However, we did not obtain an assembled TU because *bla*_TEM-1B_ amplification was mediated by IS*26*. On the other hand, an IS*26* transposon provides a direct-repeat sequence that is ideal for observing unequal crossing over because two direct repeats on sister chromatids may be incorrectly paired during replication and recombine with each other, resulting in duplication of intervening sequences (25–27). However, we demonstrated with the aid of a constructed plasmid (pUC57 and IS*26*-*bla*_KPC-2_-IS*26*) that amplification of *bla*_KPC-2_ could occur in a *recA*-deficient host (E. coli Top10), indicating that amplification is RecA-independent. Gene duplications arise through nonequal homologous recombination between long, direct-repeat sequences, such as insertion sequences previously considered to be at work in *recA*-dependent mechanisms (10). Therefore, the mechanisms underlying *bla*_KPC-2_ amplification by IS*26* require further resolution.

Tandem amplification is generally unstable and transient, making it difficult to trace. Fortunately, genome sequencing has greatly empowered strategies for assessing amplification. To illustrate, tandem repeats of ARGs flanked by IS*26* were found in the genomes of E. coli, K. pneumoniae, Acinetobacter baumannii, and Proteus mirabilis (Table S4). Furthermore, ARG repeats mediated by IS*1*, IS*5*, IS*10*, IS*CR1*, IS*CR2*, IS*1236*, IS*1216E*, IS*466*, and IS*50* (Table S4) have been detected in E. coli, K. pneumoniae, Acinetobacter baylyi, Haemophilus influenzae, *Enterococcus* spp., Salmonella typhimurium, Streptomyces coelicolor, and Raoultella planticola. These data suggested that tandem gene amplification mediated by IS occurred frequently on bacterial genomes, allowing researchers to gain insights into the pathogenic roles of transposons in the resistance development of bacteria under antibiotic stress.

10.1128/mbio.03340-21.7 TABLE S4 Examples of gene amplification mediated by mobile elements (MEs) in antibiotic resistance development. Download Table S4, XLSX file, 0.02 MB. Copyright © 2022 Wei et al. 2022 Wei et al. https://creativecommons.org/licenses/by/4.0/ This content is distributed under the terms of the Creative Commons Attribution 4.0 International license.

In summary, our work highlights *bla*_KPC-2_ amplification as key molecular underpinnings of bacterial adaptation to the sublethal challenge of single or multiple antibiotics (such as carbapenems and aminoglycosides), which is rapidly and robustly mediated by IS*26* under selective pressure. Our evidence also strongly implicates genomic plasticity (such as dynamic changes in *bla*_KPC-2_ copy numbers during antibiotic exposure) and intricate genetic rearrangement as critically important to the emergence of carbapenem hyperresistance. Overall, our work has advanced a theoretically useful perspective on identifying bacterial targets for combating the insidious threat from multidrug-resistant pathogens armed with gene amplification machinery. To help improve clinical outcomes, further investigation on novel, accessible detection measures against such under-examined pathogens are urgently needed.

## MATERIALS AND METHODS

### Bacterial strains, culture, and antibiotics.

E. coli E01-7-1, a clinical strain, was isolated from a hospital in Beijing, China, as described previously (13). E. coli Top10 was used for plasmid cloning and amplification experiments. All strains were cultured at 37°C in a lysogeny broth (LB) medium. Meropenem (MEM), tobramycin (TOB), and kanamycin (KAN) were used in the experiments (MEM, TOB, and KAN were purchased from Solarbio). The antimicrobial susceptibility of strains was determined by broth microdilution assays in cation-adjusted Mueller-Hinton broth. MIC determination was performed with a serial dilution technique in 96-well microtiter plates and interpreted in accordance with guidelines set out by the Clinical and Laboratory Standards Institute (28^th^ edition) (http://www.clsi.org/).

### Population analysis profile (PAP).

E. coli E01-7-1 was initially cultured on LB agar without antibiotics. A single colony was picked from an agar plate and pregrown in 4 mL LB medium overnight (12 h) with shaking at 200 rpm. During the antibiotic preexposure phase, bacterial cells were transferred to 4 mL LB with 0.5 and 2 μg/mL MEM or 4 mL LB without antibiotics followed by incubation at 37°C for 24 h (i.e., 12 h times two). After adjusting bacterial density to an optical density at 600 nm (OD_600_) of 1.0, plating was performed by serial dilutions on LB agar with or without various concentrations of MEM (0, 0.125, 0.25, 0.5, 1, 2, 4, 8, 16, 32, 64, 128, 256, and 512 μg/mL). CFU was enumerated after 24 h culture at 37°C. The percentage of bacteria exhibiting MEM resistance was calculated as the number of colonies growing on MEM agar plates divided by the number of bacterial colonies grown on plain LB.

### *In vitro* gene amplification experiments.

Cells cultured overnight (12 h) as above in PAP were preexposed to 2 μg/mL MEM or 4 mL Antibiotic-free LB was added to a bacterial inoculum of 5 × 10^7^ CFU/mL followed by incubation at 37°C for 24 h (with transferring once at 12 h). Cells from the two conditions were then separately transferred to a 4 mL LB medium containing various concentrations of MEM (16 μg/mL, 32 μg/mL, 64 μg/mL, 128 μg/mL, 256 μg/mL, or 512 μg/mL) with an inoculum size of 5 × 10^7^ CFU/mL. Subsequently, the strains were incubated at 37°C for 24 h during the regrowth phase. Efflux pump inhibition was performed by using phenylalanine-arginine *β*-naphthylamide (PA*β*N) as a supplement at a final concentration of 50 μM in LB. A ≥4-fold decrease in MIC in the presence of PA*β*N was considered significant. PCR was performed for targeted resistance genes and their promoter, and the PCR products were sequenced by using an ABI3730 sequencer.

Top10 E. coli strain transformed with pUC57:Tn*7094* was cultured on LB agar with 50 μg/mL KAN. A single colony was picked from an agar plate and pregrown in a 4 mL LB medium. After 12 h of incubation, bacterial cells were transferred to 4 mL LB with 1 μg/mL MEM or LB without antibiotics as a control followed by further incubation at 37°C for 24 h. Bacterial cells from the two conditions were then transferred to a 4 mL LB medium containing 1 μg/mL or 16 μg/mL MEM. The same methods were used in experiments for TOB and combination drug treatment. Each experiment was performed in triplicate in independent cultures. Bacteria were later harvested for the nitrocefin assay and RNA/DNA or plasmid extraction.

### Determination of gene copy numbers through qPCR.

Changes in gene copy numbers of *bla*_KPC-2_, *aac(3)-IIa*, *aph(3′)-Ia*, IS*26*, and *repA* were calculated through qPCR by using the 2^−ΔCt^ method to determine copy numbers against the single-copy housekeeping gene *purA*. Primers used here were the same as those for qRT-PCR (Table S5). All experiments were performed with a minimum of three biological replicates and three technical replicates per run. At least three replicates with Ct value standard deviation (SD) <0.3 were used to determine to mean Ct values.

10.1128/mbio.03340-21.8 TABLE S5 Primer name and sequences used for PCR amplification and qPCR in this study. Download Table S5, XLSX file, 0.01 MB. Copyright © 2022 Wei et al. 2022 Wei et al. https://creativecommons.org/licenses/by/4.0/ This content is distributed under the terms of the Creative Commons Attribution 4.0 International license.

Additional methods were detailed in Text S1, including *β*-lactamase enzyme activity assays, determination of transcription levels by qRT-PCR, whole-genome sequencing, and bioinformatics analysis, evaluation of amplification stability, and construction of pUC57:Tn*7094.*

10.1128/mbio.03340-21.9 TEXT S1 Additional experimental procedures. Download Text S1, DOCX file, 0.02 MB. Copyright © 2022 Wei et al. 2022 Wei et al. https://creativecommons.org/licenses/by/4.0/ This content is distributed under the terms of the Creative Commons Attribution 4.0 International license.

### Availability of data.

All sequence data are available for direct download at the China National Microbiology Data Center (NMDC) with the following accession number: NMDCX0000112.

## References

[1] Band VI, Hufnagel DA, Jaggavarapu S, Sherman EX, Wozniak JE, Satola SW, Farley MM, Jacob JT, Burd EM, Weiss DS. 2019. Antibiotic combinations that exploit heteroresistance to multiple drugs effectively control infection. Nat Microbiol 4:1627–1635. doi:10.1038/s41564-019-0480-z. 31209306 PMC7205309

[2] Nordmann P, Cuzon G, Naas T. 2009. The real threat of *Klebsiella pneumoniae* carbapenemase-producing bacteria. Lancet Infect Dis 9:228–236. doi:10.1016/S1473-3099(09)70054-4. 19324295

[3] Adams-Sapper S, Nolen S, Donzelli GF, Lal M, Chen K, Justo da Silva LH, Moreira BM, Riley LW. 2015. Rapid induction of high-level carbapenem resistance in heteroresistant KPC-producing *Klebsiella pneumoniae*. Antimicrob Agents Chemother 59:3281–3289. doi:10.1128/AAC.05100-14. 25801565 PMC4432212

[4] Weisenberg SA, Morgan DJ, Espinal-Witter R, Larone DH. 2009. Clinical outcomes of patients with *Klebsiella pneumoniae* carbapenemase-producing *K. pneumoniae* after treatment with imipenem or meropenem. Diagn Microbiol Infect Dis 64:233–235. doi:10.1016/j.diagmicrobio.2009.02.004. 19345034 PMC2764245

[5] Hirsch EB, Tam VH. 2010. Detection and treatment options for *Klebsiella pneumoniae* carbapenemases (KPCs): an emerging cause of multidrug-resistant infection. J Antimicrob Chemother 65:1119–1125. doi:10.1093/jac/dkq108. 20378670

[6] Anderson KF, Lonsway DR, Rasheed JK, Biddle J, Jensen B, McDougal LK, Carey RB, Thompson A, Stocker S, Limbago B, Patel JB. 2007. Evaluation of methods to identify the *Klebsiella pneumoniae* carbapenemase in Enterobacteriaceae. J Clin Microbiol 45:2723–2725. doi:10.1128/JCM.00015-07. 17581941 PMC1951220

[7] Nicoloff H, Hjort K, Levin BR, Andersson DI. 2019. The high prevalence of antibiotic heteroresistance in pathogenic bacteria is mainly caused by gene amplification. Nat Microbiol 4:504–514. doi:10.1038/s41564-018-0342-0. 30742072

[8] Cui X, Shan B, Zhang X, Qu F, Jia W, Huang B, Yu H, Tang Y-W, Chen L, Du H. 2020. Reduced Ceftazidime-Avibactam Susceptibility in KPC-Producing *Klebsiella pneumoniae* From Patients Without Ceftazidime-Avibactam Use History - A Multicenter Study in China. Front Microbiol 11:1365. doi:10.3389/fmicb.2020.01365. 32655534 PMC7324628

[9] Sun D, Rubio-Aparicio D, Nelson K, Dudley MN, Lomovskaya O. 2017. Meropenem-Vaborbactam Resistance Selection, Resistance Prevention, and Molecular Mechanisms in Mutants of KPC-Producing *Klebsiella pneumoniae*. Antimicrob Agents Chemother 61. doi:10.1128/AAC.01694-17. PMC5700310 29038260

[10] Sandegren L, Andersson DI. 2009. Bacterial gene amplification: implications for the evolution of antibiotic resistance. Nat Rev Microbiol 7:578–588. doi:10.1038/nrmicro2174. 19609259

[11] Liu X, Liu F, Ding S, Shen J, Zhu K. 2020. Sublethal Levels of Antibiotics Promote Bacterial Persistence in Epithelial Cells. Adv Sci (Weinh) 7:1900840. doi:10.1002/advs.201900840. 32999821 PMC7509632

[12] Gullberg E, Albrecht LM, Karlsson C, Sandegren L, Andersson DI. 2014. Selection of a multidrug resistance plasmid by sublethal levels of antibiotics and heavy metals. mBio 5:e01918-14. doi:10.1128/mBio.01918-14. 25293762 PMC4196238

[13] Gong L, Tang N, Chen D, Sun K, Lan R, Zhang W, Zhou H, Yuan M, Chen X, Zhao X, Che J, Bai X, Zhang Y, Xu H, Walsh TR, Lu J, Xu J, Li J, Feng J. 2020. A Nosocomial Respiratory Infection Outbreak of Carbapenem-Resistant *Escherichia coli* ST131 With Multiple Transmissible bla KPC-2 Carrying Plasmids. Front Microbiol 11:2068. doi:10.3389/fmicb.2020.02068. 33042037 PMC7516988

[14] Zhao L, Liu Y, Kou Z, Bayasi A, Cai H, Zhang C, Wang Q, Li Y, Fang Y. 2011. Improved RP-HPLC method to determine biapenem in human plasma/urine and its application to a pharmacokinetic study. Arzneimittelforschung 61:197–204. doi:10.1055/s-0031-1296189. 21528646

[15] Andersson DI, Nicoloff H, Hjort K. 2019. Mechanisms and clinical relevance of bacterial heteroresistance. Nat Rev Microbiol 17:479–496. doi:10.1038/s41579-019-0218-1. 31235888

[16] Lazar V, Kishony R. 2019. Transient antibiotic resistance calls for attention. Nat Microbiol 4:1606–1607. doi:10.1038/s41564-019-0571-x. 31541208

[17] Andersson DI, Hughes D. 2014. Microbiological effects of sublethal levels of antibiotics. Nat Rev Microbiol 12:465–478. doi:10.1038/nrmicro3270. 24861036

[18] Kitchel B, Rasheed JK, Endimiani A, Hujer AM, Anderson KF, Bonomo RA, Patel JB. 2010. Genetic factors associated with elevated carbapenem resistance in KPC-producing *Klebsiella pneumoniae*. Antimicrob Agents Chemother 54:4201–4207. doi:10.1128/AAC.00008-10. 20660684 PMC2944623

[19] Nodari CS, Ribeiro VB, Barth AL. 2015. Imipenem heteroresistance: high prevalence among Enterobacteriaceae *Klebsiella pneumoniae* carbapenemase producers. J Med Microbiol 64:124–126. doi:10.1099/jmm.0.081869-0. 25351714

[20] Yigit H, Queenan AM, Rasheed JK, Biddle JW, Domenech-Sanchez A, Alberti S, Bush K, Tenover FC. 2003. Carbapenem-resistant strain of *Klebsiella oxytoca* harboring carbapenem-hydrolyzing beta-lactamase KPC-2. Antimicrob Agents Chemother 47:3881–3889. doi:10.1128/AAC.47.12.3881-3889.2003. 14638498 PMC296202

[21] Wang XC, Lei CW, Kang ZZ, Zhang Y, Wang H. N. 2019. IS*26*-mediated genetic rearrangements in *Salmonella* genomic island 1 of *Proteus mirabilis*. Front Microbiol 10:2245. doi:10.3389/fmicb.2019.02245. 31608048 PMC6769106

[22] Hawkey J, Le Hello S, Doublet B, Granier SA, Hendriksen RS, Fricke WF, Ceyssens P, Gomart C, Billman-Jacobe H, Holt KE, Weill F. 2019. Global phylogenomics of multidrug-resistant *Salmonella enterica* serotype Kentucky ST198. Microb Genom 5:e000269. doi:10.1099/mgen.0.000269. PMC6700661 31107206

[23] He DD, Zhao SY, Wu H, Hu GZ, Zhao JF, Zong ZY, Pan YS. 2019. Antimicrobial resistance-encoding plasmid clusters with heterogeneous MDR regions driven by IS*26* in a single *Escherichia coli* isolate. J Antimicrob Chemother 74:1511–1516. doi:10.1093/jac/dkz044. 30820562

[24] Hubbard ATM, Mason J, Roberts P, Parry CM, Corless C, van Aartsen J, Howard A, Bulgasim I, Fraser AJ, Adams ER, Roberts AP, Edwards T. 2020. Piperacillin/tazobactam resistance in a clinical isolate of *Escherichia coli* due to IS*26*-mediated amplification of *bla*_TEM-1B_. Nat Commun 11:4915. doi:10.1038/s41467-020-18668-2. 33004811 PMC7530762

[25] Andersson DI, Jerlstrom-Hultqvist J, Nasvall J. 2015. Evolution of new functions de novo and from preexisting genes. Cold Spring Harb Perspect Biol 7:a017996. doi:10.1101/cshperspect.a017996. 26032716 PMC4448608

[26] Kugelberg E, Kofoid E, Andersson DI, Lu Y, Mellor J, Roth FP, Roth JR. 2010. The tandem inversion duplication in *Salmonella enterica*: selection drives unstable precursors to final mutation types. Genetics 185:65–80. doi:10.1534/genetics.110.114074. 20215473 PMC2870977

[27] Smith GP. 1976. Evolution of repeated DNA sequences by unequal crossover. Science 191:528–535. doi:10.1126/science.1251186. 1251186

