# Erratum for Wei et al., “IS*26* Veers Genomic Plasticity and Genetic Rearrangement toward Carbapenem Hyperresistance under Sublethal Antibiotics”

**DOI:** 10.1128/mbio.00414-22

**Published:** 2022-03-07

**Authors:** Da-Wei Wei, Nai-Kei Wong, Yuqin Song, Gang Zhang, Chao Wang, Juan Li, Jie Feng

**Affiliations:** a State Key Laboratory of Microbial Resources, Institute of Microbiology, Chinese Academy of Sciences, Beijing, China; b College of Life Science, University of Chinese Academy of Sciences, Beijing, China; c Clinical Pharmacology Section, Department of Pharmacology, Shantou University Medical College, Shantou, China; d State Key Laboratory of Infectious Disease Prevention and Control, National Institute for Communicable Disease Control and Prevention, Chinese Centers for Disease Control and Prevention, Changping, Beijing, China

## ERRATUM

Volume 13, no. 1, e03340-21, 2022, https://doi.org/10.1128/mBio.03340-21. There was an error in the title of the original publication. The correct title is shown in this erratum.

