# Supplementary figures and images for: IS26 Veers Genomic Plasticity and Genetic Rearrangement toward Carbapenem Hyperresistance under Sublethal Antibiotics
Source: mBio. 2022 Feb 8;13(1):e03340-21. doi: 10.1128/mbio.03340-21 (PMC8822349; doi:10.1128/mbio.03340-21)

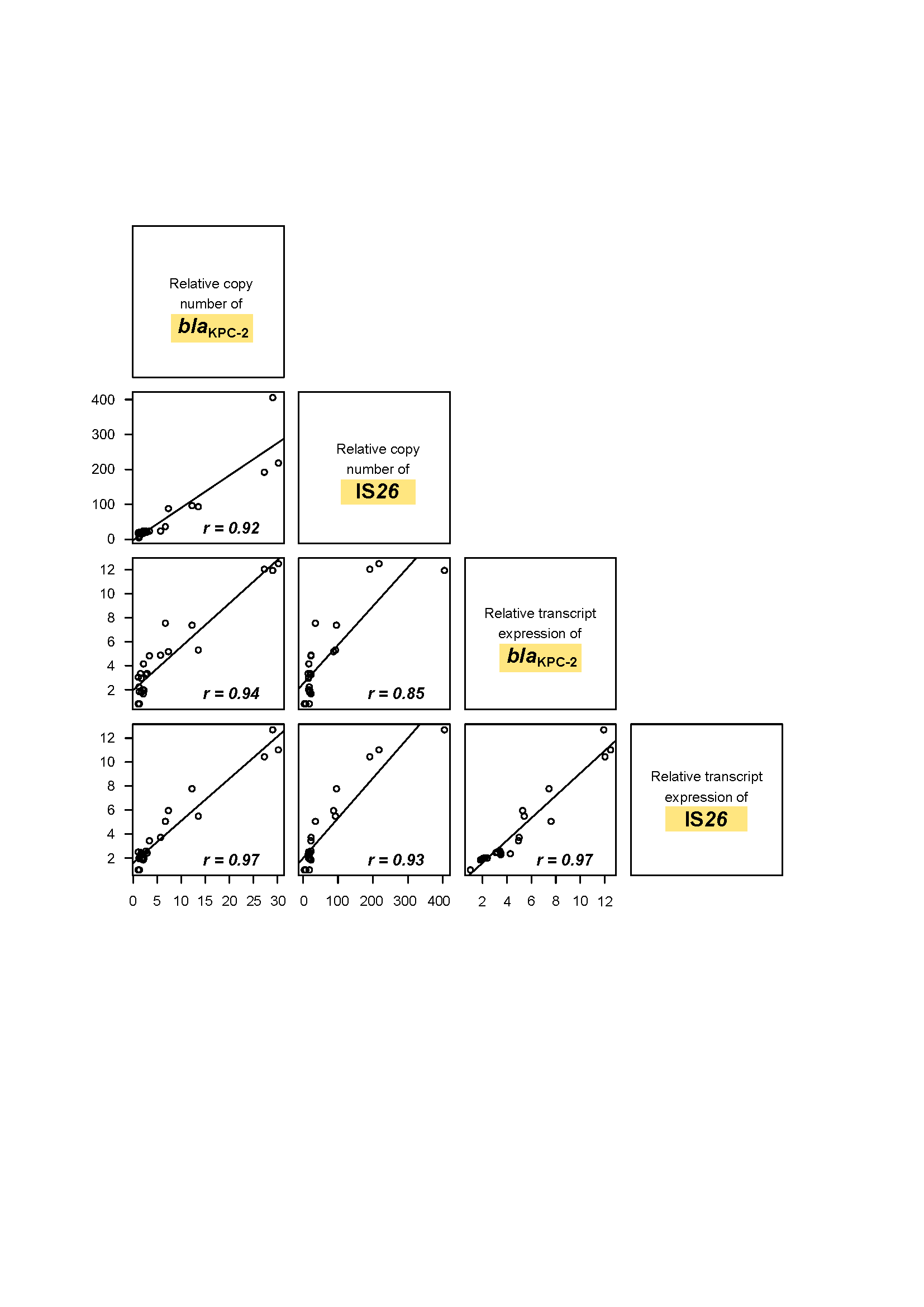

Supplement: FIG S2 [file mbio.03340-21-sf002.tif]

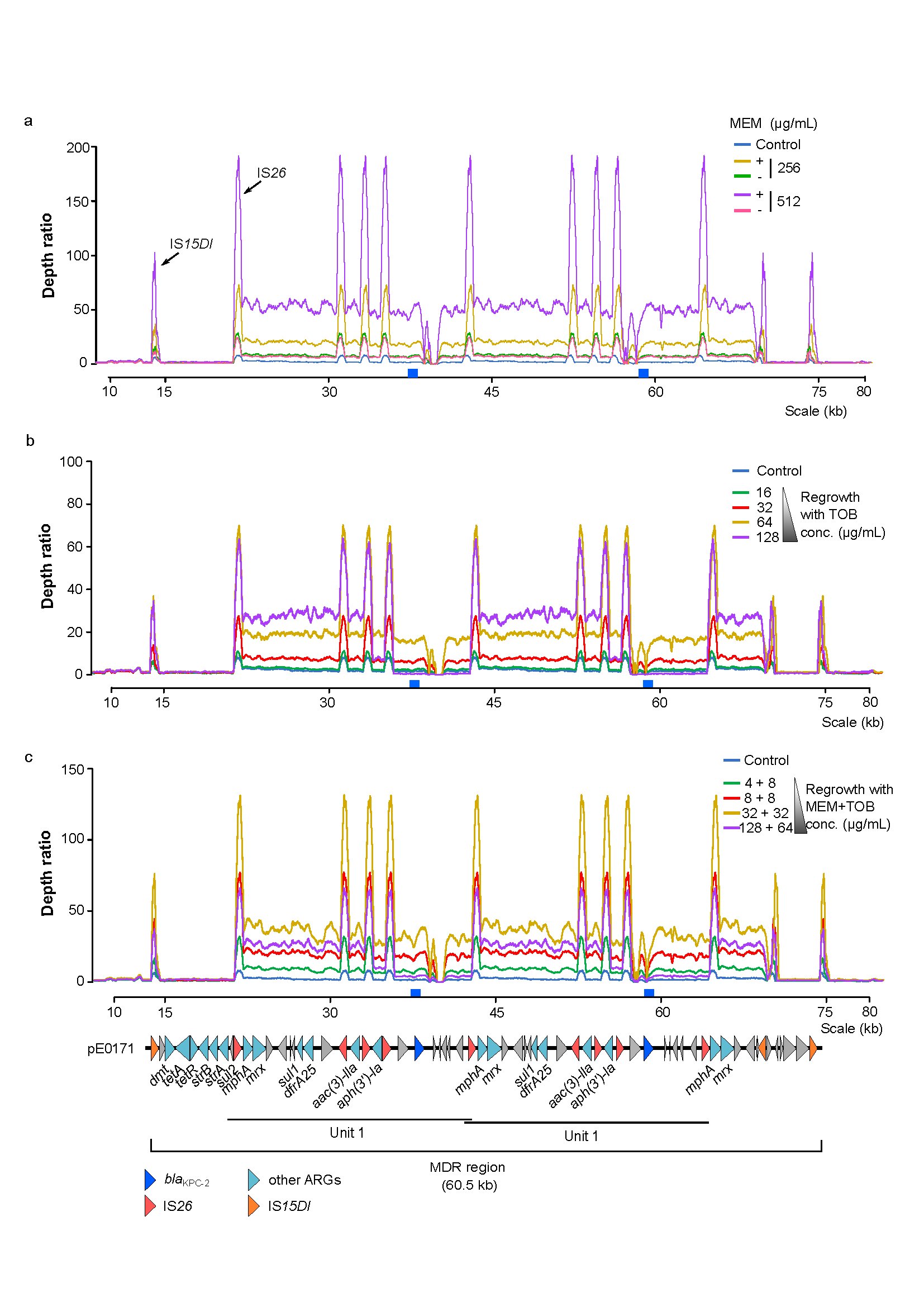

Supplement: FIG S3 [file mbio.03340-21-sf003.tif]
