# Supplementary material for: IS26 Veers Genomic Plasticity and Genetic Rearrangement toward Carbapenem Hyperresistance under Sublethal Antibiotics
Source: mBio. 2022 Feb 8;13(1):e03340-21. doi: 10.1128/mbio.03340-21 (PMC8822349; doi:10.1128/mbio.03340-21)
Supplement: TEXT S1 [file mbio.03340-21-s0001.docx]

**Supplementary methods**

***β*-lactamase enzyme activity assays.** *β*-lactam hydrolysis was evaluated by using a colorimetric nitrocefin assay. Bacterial cells harvested by the abovementioned method were washed twice with phosphate-buffered saline (PBS, pH 7.4), centrifuged at 12,000 rpm and 4°C for 5 min. Then, the pellet was resuspended in PBS, adjusted to OD_600 nm_ = 0.2. From the suspension, 5 mL was sampled and lysed via sonication on ice for 10 min. Subsequently, the supernatant was collected through centrifugation and diluted 5× in PBS. Then, 10 μL of 0.5 μg/mL nitrocefin was added to 90 μL of the diluted cell lysate. The assay is based on the hydrolysis of the substrate Nitrocefin, a chromogenic cephalosporin, that results in the generation of a colored product (detectable at OD_450_ nm), which is directly proportional to the amount of beta-lactamase activity. Absorbance at OD_450 nm_ was measured every 20 s for 40 min by means of a BioTek Synergy H4 Hybrid multimode microplate reader. The assay was performed in three biological replicates and three technical replicates.

**Determination of transcription levels by qRT-PCR.** Total RNA was extracted with a FastPure Cell/Tissue Total RNA Isolation Kit V2 (Vazyme Biotech Co., Nanjing, China). Reverse transcription was performed by using HiScript II reverse transcriptase (Vazyme Biotech Co.). A total of 1 μg RNA was used for reverse transcription with random hexamers (50 ng) and resultant cDNA was diluted 10-fold for qPCR. Primers targeting the housekeeping gene *purA*, *bla*_KPC-2_, IS*26*, and plasmid-borne *repA* were designed by using an online tool (<https://sg.idtdna.com/>) (Table S5). Each qPCR reaction was performed with ChamQ Universal SYBR qPCR master mix (Vazyme Biotech Co.) by using the Applied Biosystems ViiA7 system. Relative expression was calculated by using the 2^−ΔΔCt^ method. All experiments were performed with a minimum of three biological replicates and three technical replicates per run. At least three replicates with Ct value SD <0.3 were used to determine mean Ct values.

**Whole-genome sequencing and bioinformatics analysis.** Whole-genome sequencing (WGS) was performed with DNA extracts of relevant cultures on Illumina NovaSeq 6000 (Illumina, San Diego, CA, USA) and PromethION (Oxford Nanopore Technologies, Oxford, UK) platforms. The former platform was used to generate 2× 150-bp pair-ended reads of high quality for each sample, while the latter was used to generate sufficiently long reads to delineate the complete genome of strain E0171 grown under MEM concentration 512 μg/mL for analysis on complex structures or regions. Plasmids containing Tn*7094* were extracted for Nanopore sequencing.

SPAdes v3.13.0 was used for *de novo* assembly from Illumina data to obtain draft genomes. Flye v2.6 was used for *de novo* assembly based on Nanopore reads. Then, correction and genome polishing were performed through Racon v1.4.7. NextPolish v1.4.7 and Pilon v1.23 were used to obtain complete genomes from Illumina reads. We used SOAPaligner v2.20 to map out clean read sequences from each sample for sequencing of the pE0171_KPC plasmid. The command soap.coverage (downloaded from <https://github.com/gigascience/bgi-soap2/tree/master/tools/soap>.coverage) was employed to independently calculate mapping depths of each sample. Mapping results were normalized to mean depths of the *repA* gene, which were then plotted out in R (v3.5.1). Subsequently, relative copy numbers of *bla*_KPC-2_ and IS*26* were calculated by using mean depths of these two genes to differentiate sequencing results for *repA* gene and *purA* gene.

PromethION long-read sequences for E01-7-1 in fastq format were first transformed into fasta format through an in-house Perl script. Then, fasta file was aligned to reference sequences of *bla*_KPC-2_ and the entire MDR region by using blastn. Reads with more than two *bla*_KPC-2_ sequences or containing the bulk of the MDR region were filtered out with cutoffs of coverage ≥90% and identity ≥90%. Several representative reads were selected and a schematic map of their linear comparisons was created by using EasyFig (v2.2.2) and adjusted through Inkscape (v0.92). Reads of plasmid pUC57: Tn*7094* were filtered in a similar manner. Data are available at the China National Microbiology Data Center (NMDC) under the accession number: NMDCX0000112, Supplement 1 is the original data of complete genome of strain E01-7-1 at 512 μg/ mL meropenem; Supplement 2 is the original data of pUC57:Tn*7094*; Supplement 3 and 4 are the filtered reads; Supplement 5 is the sequence of pUC57: Tn*7094* we constructed; Supplement 6 is the clean data of that used for mapping analysis.

**Evaluation of amplification stability.** Three independently replicated cultures grown with MEM at high concentrations (128 μg/mL, 256 μg/mL and 512 μg/mL) were individually diluted 100× with fresh LB medium without antibiotics. After growth for 24 h (with transferring once at 12 h) at 37°C with agitation at 200 rpm, total DNA was extracted from harvested bacteria. Relative copy numbers of *bla*_KPC-2_ and IS*26* were determined through qPCR and Illumina sequencing. Stability of copy numbers was evaluated through a comparison of copy numbers for cultures grown at high MEM concentrations.

**Construction of pUC57: Tn*7094.*** The transposon IS*26*-*bla*_KPC-2_-IS*26* was designated as Tn*7094* according to Isfinder (<https://www-is.biotoul.fr/index.php>). It was synthesized by Sangon Biotech (Shanghai, China). Then, Tn*7094* was subcloned into the plasmid pUC57 upon digestioin with EcoRI and BamHI. *E. coli* Top10 was transformed with the resultant recombinant plasmids and clones were selected on LB agar plates, supplemented with 50 μg/mL KAN. Transformants were validated through restriction enzyme digestion, DNA gel electrophoresis and sequencing. The successfully validated recombinant plasmid was designated pUC57: Tn*7094* (6,024 bp).
